# MBD2 mediates renal cell apoptosis via activation of Tox4 during rhabdomyolysis‐induced acute kidney injury

**DOI:** 10.1111/jcmm.16207

**Published:** 2021-03-25

**Authors:** Tianshi Sun, Qing Liu, Yifan Wang, Youwen Deng, Dongshan Zhang

**Affiliations:** ^1^ Department of Spine Surgery The Third Xiangya Hospital of Central South University Changsha China; ^2^ Department of Emergency Medicine Second Xiangya Hospital of Central South University Changsha China; ^3^ Emergency Medicine and Difficult Diseases Institute Second Xiangya Hospital of Central South University Changsha China; ^4^ Department of Nephrology Second Xiangya Hospital of Central South University Changsha China

**Keywords:** AKI, apoptosis, MBD2, rhabdomyolysis, Tox4

## Abstract

Our study investigated the role of Methyl‐CpG–binding domain protein 2 (MBD2) in RM‐induced acute kidney injury (AKI) both in vitro and in vivo. MBD2 was induced by myoglobin in BUMPT cells and by glycerol in mice. MBD2 inhibition via MBD2 small interfering RNA and MBD2‐knockout (KO) attenuated RM‐induced AKI and renal cell apoptosis. The expression of TOX high mobility group box family member 4 (Tox4) induced by myoglobin was markedly reduced in MBD2‐KO mice. Chromatin immunoprecipitation analysis indicated that MBD2 directly bound to CpG islands in the Tox4 promoter region, thus preventing promoter methylation. Furthermore, siRNA inhibition of Tox4 attenuated myoglobin‐induced apoptosis in BUMPT cells. Finally, MBD2‐KO mice exhibited glycerol‐induced renal cell apoptosis by inactivation of Tox4. Altogether, our results suggested that MBD2 plays a role in RM‐induced AKI via the activation of Tox4 and represents a potential target for treatment of RM‐associated AKI.

## 1 INTRODUCTION

Rhabdomyolysis (RM) is a life‐threatening condition, characterized by skeletal muscle tissue damage and leakage of intracellular components into the bloodstream. Traumatic RM occurs in the case of crush syndrome,^1^, ^2^ which is a medical condition that occurs due to crushing skeletal injury in victims of natural disasters (eg earthquakes), accidents and wars.^3^, ^4^ One of the most common complications of RM is acute kidney injury (AKI), and 10% of all AKI patients present with RM.^5^ AKI is a clinical disorder with high mortality rates.^6^ It is known that the renal toxicity of myoglobin plays a crucial role in RM‐induced AKI via increased oxidative stress, cast formation, inflammation and apoptosis.^7^ Although numerous studies have focused on RM‐induced AKI,^7^, ^8^, ^9^, ^10^ the underlying mechanisms remain largely unknown, thus resulting in non‐specific therapy.

Previous research has shown that DNA methylation occurs in regions containing a high density of CpG dinucleotides, which is typically thought to result in epigenetic modification related to transcriptional repression; however, the role and traditional interpretation of DNA methylation has been recently challenged.^11^ Based on different methylation patterns, it has been reported that DNA methylation may play a role in both transcriptional activation and silencing. Furthermore, accumulating evidence suggests that DNA methylation is associated with AKI.^5^, ^12^, ^13^ However, the role of DNA methylation in RM‐induced AKI may vary based on differential regulation of methylation and the type of experimental model used. In mice that are injected with glycerol in their bilateral hindlimb muscles,^14^ RM‐induced AKI is characterized by renal cortical necrosis, cast formation and inflammatory infiltration.^15^, ^16^ Apoptosis is a key phenomenon in glycerol‐induced AKI, which may, in turn, be regulated by other cellular pathways.^16^, ^17^, ^18^ Therefore, it is important to gain a holistic understanding of the mechanisms underlying RM‐induced AKI to better design targeted treatment therapies.

Here, we aimed to study the role of DNA methylation in AKI by analysing the function of Methyl‐CpG–binding domain protein 2 (MBD2), a key methylated protein reader, in RM‐induced AKI, both in vitro using a mouse proximal tubule‐derived cell line (BUMPT) and in vivo using C57BL/6 mice.

## 2 MATERIALS AND METHODS

### 2.1 Reagents and antibodies

Antibodies were purchased from the following sources: MBD2 from Abcam (Cambridge, UK); β‐Tubulin from Santa Cruz Biotechnology (Santa Cruz, CA, USA); Caspase‐3, cleaved caspase‐3 from Cell Signaling Technology (Danvers, MA, USA); Tox4 (PA5‐53653) from Thermo Fisher Scientific (Waltham, MA, USA). Myoglobin and ascorbic acid were purchased from Sigma‐Aldrich (St. Louis, MO, USA). The *Tox4* promoter containing the methylated target sequences of MBD2 was subcloned into a CpG‐free pCpGI luciferase reporter vector (Invitrogen Biotechnology, Shanghai, China). Construction of methylation promoter of Tox4 CpG‐free pCpGI luciferase reporter, MBD2 and mtMBD2 (the deletion of the methylated DNA‐binding domain) plasmids as described previously.^36^, ^37^ Small interfering RNAs (siRNAs) against *MBD2* and *Tox4* were synthesized by RUIBO Biology Co., Ltd. (Guangdong, Guangzhou, China), as previously described.^26^

### 2.2 Animal model of RM‐induced AKI

MBD2 knockout (MBD2‐KO) mice were obtained from Cyagen Biosciences Co., Ltd (Guangzhou, China). Male MBD2‐KO mice aged 10‐12 weeks were injected in the bilateral hindlimb skeletal muscles with a dose of 8.0 mL/kg glycerol (50% v/v in sterile saline); the control group was injected with an equal volume of normal saline. Littermate male MBD2‐wild‐type (WT) mice were treated using the same method mentioned above. Animal experimental protocols were approved by the Care and Use of Laboratory Animals Institutional Committee from Second Xiangya Hospital, China. The mice were housed at stable room temperature in a 12‐hour light/dark cycle and provided adequate supplies of standard rodent chow and water.

### 2.3 Cell culture and treatments

BUMPT cells were cultured in Dulbecco's modified Eagle's medium with 10% foetal bovine serum, 0.5% penicillin (Thermo Fisher Scientific), and streptomycin, and then maintained in a 5%‐CO2 incubator at 37°C. Both myoglobin and ascorbic acid were then added to the medium at a final concentration of 200 mM (3.6 mg/mL) and 2 mM, respectively. Ascorbic acid reduces myoglobin to a ferrous status,^29^ and the cytotoxic ferrous myoglobin causes renal tubular injury. Chromatin immunoprecipitation (ChIP) experiments were performed using anti‐MBD2, according to the ChIP kit assay procedure (Millipore, Burlington, MA, USA). Immunoprecipitated DNA was amplified by polymerase chain reaction (PCR) using the following primers that bound to CpG islands in the binding of promoter of Tox4 promoter:

F1:5′‐GGAGGGTTGGGGTTTTAGTA‐3′; R1:5′‐AACATCAACAACTTTTACTCACCTC‐3′; F2:5′‐GGAGGGTTGGGGTTTTAGTA‐3′; R2:5′‐CAACATCAACAACTTTTACTCACCT‐3′; F3:5′‐GGAGGGTTGGGGTTTTAGTA‐3′, R3:5′‐CCAACATCAACAACTTTTACTCAC‐3′; F4:5′‐GGAGGGTTGGGGTTTTAGTA‐3′; R4:5′‐ACATCAACAACTTTTACTCACCTC‐3′; F5:5′‐GGAGGGTTGGGGTTTTAGTA‐3′; R5:5′‐CCCAACATCAACAACTTTTACTC‐3.

Transcriptional activation activity of Tox4 was measured using the luciferase kit (Promega, Madison, WI, USA), as described previously.^26^, ^30^, ^31^

### 2.4 Analysis of apoptosis

Apoptosis in kidney tissues was analysed by terminal deoxynucleotidyl transferase‐mediated digoxigenin‐deoxyuridine nick‐end labelling (TUNEL) assay using the In Situ Cell Death Detection Kit from Roche Diagnostics (Indianapolis, IN). Apoptosis in cell cultures was analysed by flow cytometry (FCM), as previously described.^26^, ^31^, ^35^

### 2.5 Methylated CpG‐DNA immunoprecipitation

Immunoprecipitation of methylated CpG‐DNA was performed, as described previously (Zymo Research, Irvine, CA, USA).^32^ Briefly, sheared DNA was used for methylated CpG immunoprecipitation, and the methylated DNA was analysed by PCR analysis using an ABI Onestepplus real‐time PCR system.

### 2.6 Histology, immunohistochemistry and immunoblot analyses

Haematoxylin and eosin (H&E) staining was used for histological analysis. The tubular damage scores were assessed according to the percentage of damaged tubules, as described previously.^26^ TUNEL assay was used for detecting renal cell apoptosis, and the percentage of the total number of TUNEL‐positive cells were calculated in 10‐20 microscopic fields that were randomly selected per tissue section.^33^ Immunohistochemical staining and image analysis of MBD2 were performed according to previously described methods.^34^ Lysates of kidney tissues and BUMPT cells were subjected to sodium dodecyl sulphate‐polyacrylamide gel electrophoresis and immunoblotting was performed using MBD2, Tox4, Caspase‐3, cleaved caspase‐3 and β‐Tubulin antibodies following standard procedures.

### 2.7 Statistical analysis

Data were expressed as mean ± standard deviation (SD). Two‐group comparisons were made using 2‐tailed Student *t* tests. Multiple group data were evaluated using one‐way analysis of variance. *P* < .05 was considered statistically significant.

## 3 RESULTS

### 3.1 MBD2 expression was induced by RM in BUMPT cells and C57BL/6 mice

**FIGURE 1:**
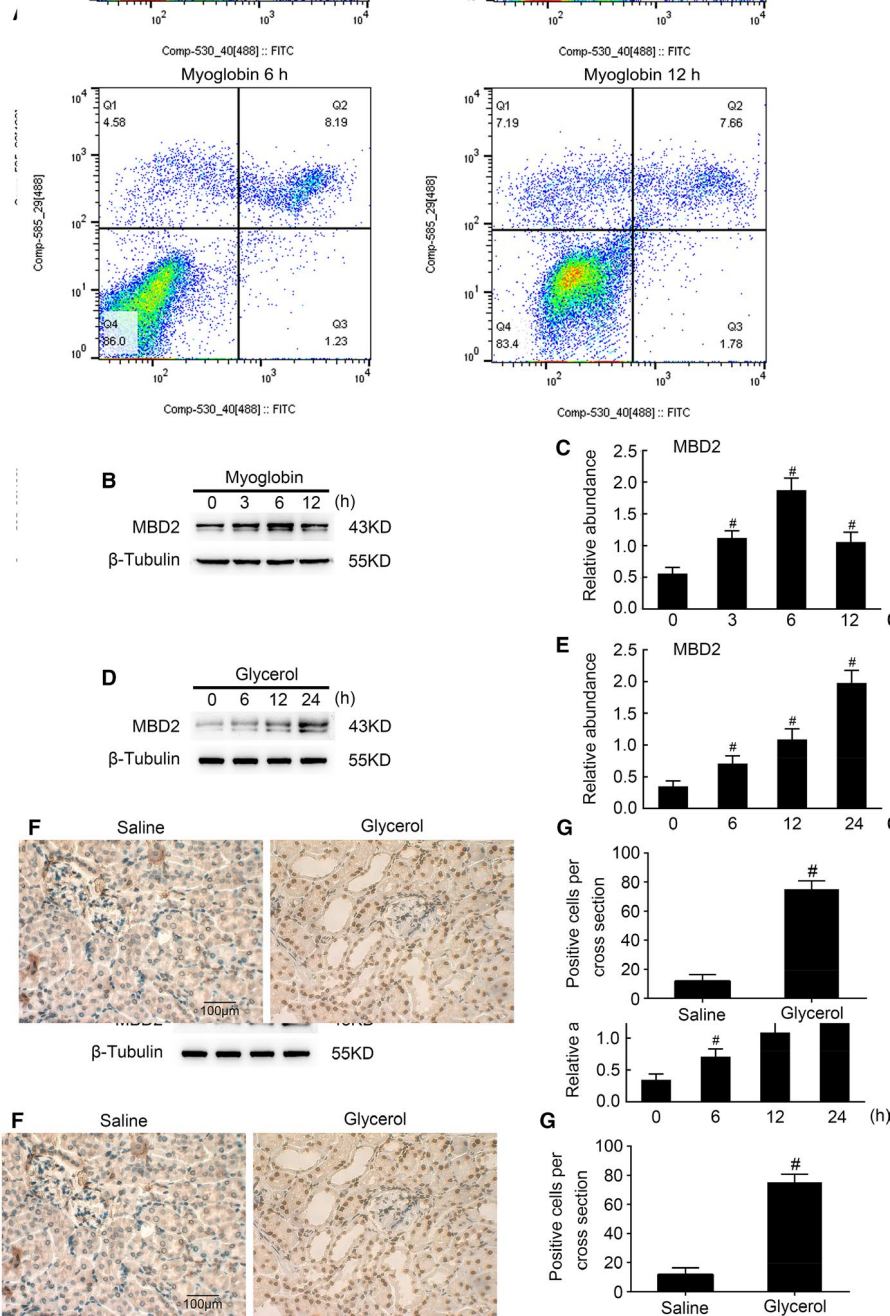
Rhabdomyolysis induced the expression of MBD2 in both BUMPT cells and mice kidneys. BUMPT cells were administered with 3.6 mg/mL ferrous myoglobin and analysed for 0‐12 h and male C57BL/6 mice were injected with 8.0 mL/kg glycerol and analysed for 0‐24 h, respectively. A, Flow cytometry analysis of apoptosis in myoglobin‐treated BUMPT cells. B and D, Whole protein lysates from cells and kidney tissues were gathered for Western blot analysis of MBD2 expression at the indicated time points. C and E, Densitometric ratios of MBD2/β‐Tubulin. F and G, Immunohistochemical staining and the calculation of MBD2 expression. Scale bar, 100 μM. Data are expressed as mean ± SD (n = 6). ^#^ *P* < .05 vs 0 h group or Saline group. Original magnification, 400× in F

Using Western blotting, we investigated the expression of MBD2 induced by RM‐associated AKI at different time points in BUMPT cells treated with ferrous myoglobin and renal tissues of C57BL/6 mice injected with glycerol. The flow cytometry analysis showed that myoglobin induced the BUMPT cell apoptosis. As shown in Figure 1B‐E, MBD2 expression levels gradually increased in BUMPT cells and renal tissues over time compared with baseline. Interestingly, in vitro MBD2 expression peaked at the 6 hours time point (Figure 1A,B). Although MBD2 levels decreased thereafter, they remained higher than pre‐treatment levels at 12 hours after myoglobin treatment. Furthermore, immunohistochemical analysis of the renal tissues of C57BL/6 mice injected with glycerol confirmed that MBD2 expression was mainly localized in the tubular cell nuclei (Figure 1F,G). Altogether, the results indicated that MBD2 expression levels were up‐regulated both in vitro and in vivo during RM‐associated AKI.

### 3.2 Renal dysfunction, tubular damage and cell apoptosis are attenuated in MBD2‐KO mice during RM‐induced AKI

**FIGURE 2:**
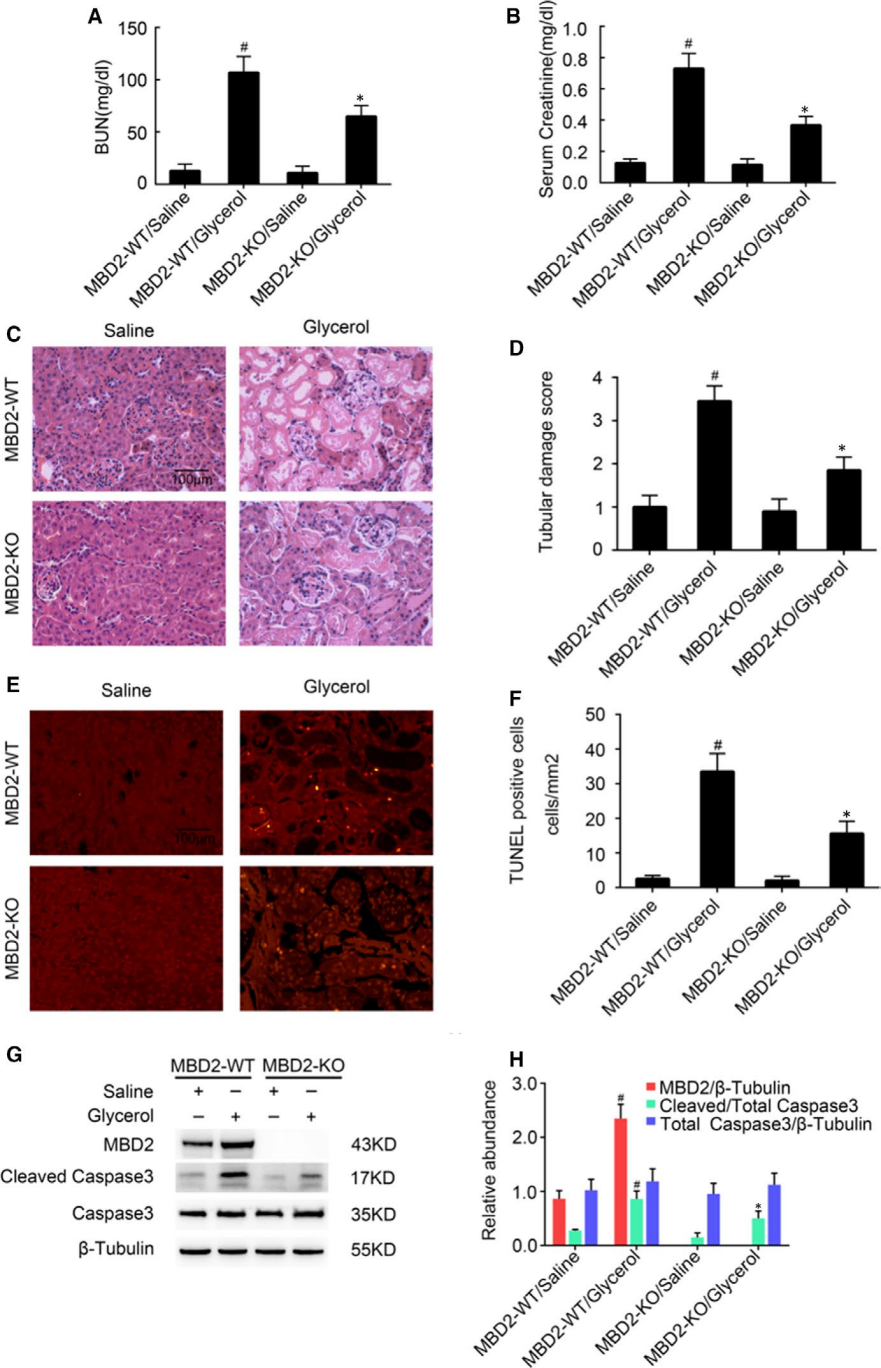
Amelioration of glycerol‐induced acute kidney injury and renal cell apoptosis in MBD2‐knockout (KO) mice. The MBD2‐KO and wild‐type (WT) littermate mice were injected with 8.0 mL/kg glycerol or saline as control for 24 h. A and B, Serum creatinine and blood urea nitrogen (BUN) were used for assessing renal function. C, The renal tissue sections were stained with haematoxylin & eosin (H&E). D, Tubular damage score in glycerol‐treated kidney cortical tissues. E, Apoptosis was analysed using the TUNEL assay. F, The calculation of TUNEL‐positive cells. G, Western blot analysis of MBD2, Caspase‐3, cleaved caspase‐3, and β‐Tubulin expression. H, Densitometric ratios of MBD2, Caspase‐3, cleaved caspase‐3 and β‐Tubulin. Scale bar, 100 μM. Data are expressed as mean ± SD (n = 6). ^#^ *P* < .05 vs Saline group, *P < .05 vs MBD2‐WT/glycerol group. Original magnification, 400× in A and 200× in E

We injected the MBD2‐KO and WT littermate mice with glycerol and analysed the effects after 24 hours. Glycerol injections induced renal failure in WT mice, as indicated by the increased levels of blood urea nitrogen (BUN) and creatinine compared with control. However, in MBD2‐KO mice, the increase in BUN and creatinine levels was lower compared with that in MBD2‐WT mice (Figure 2A,B). H&E staining also confirmed that the deletion of MBD2 markedly reduced tubular damage in the cortex of the kidney in glycerol‐injected mice (Figure 2C). Additionally, glycerol‐injected MBD2‐KO mice showed a lower tubular damage score of 1.9 compared with glycerol‐injected WT mice that showed a score of 3.5 (Figure 2D). Previous results have indicated that apoptosis plays a key role in the progression of RM‐induced AKI.^16^, ^17^, ^19^, ^20^, ^21^ To investigate whether MBD2 promoted renal cell apoptosis and, therefore, tubular cell damage, we performed TUNEL staining to assess apoptosis in WT and MBD2‐KO renal tissues. We observed increased apoptosis in the kidney cells of glycerol‐injected WT mice compared with those of MBD2‐KO mice (Figure 2E), and this increase was also reflected by the increased number of TUNEL‐positive cells in WT mice compared with MBD2‐KO mice (Figure 2F). Moreover, Western blotting showed that the levels of cleaved caspase‐3 were markedly lower in MBD2‐KO cells than in WT cells (Figure 2G), which was also supported by densitometric quantitation (Figure 2H).

### 3.3 MBD2 mediated myoglobin‐induced apoptosis in BUMPT cells

**FIGURE 3:**
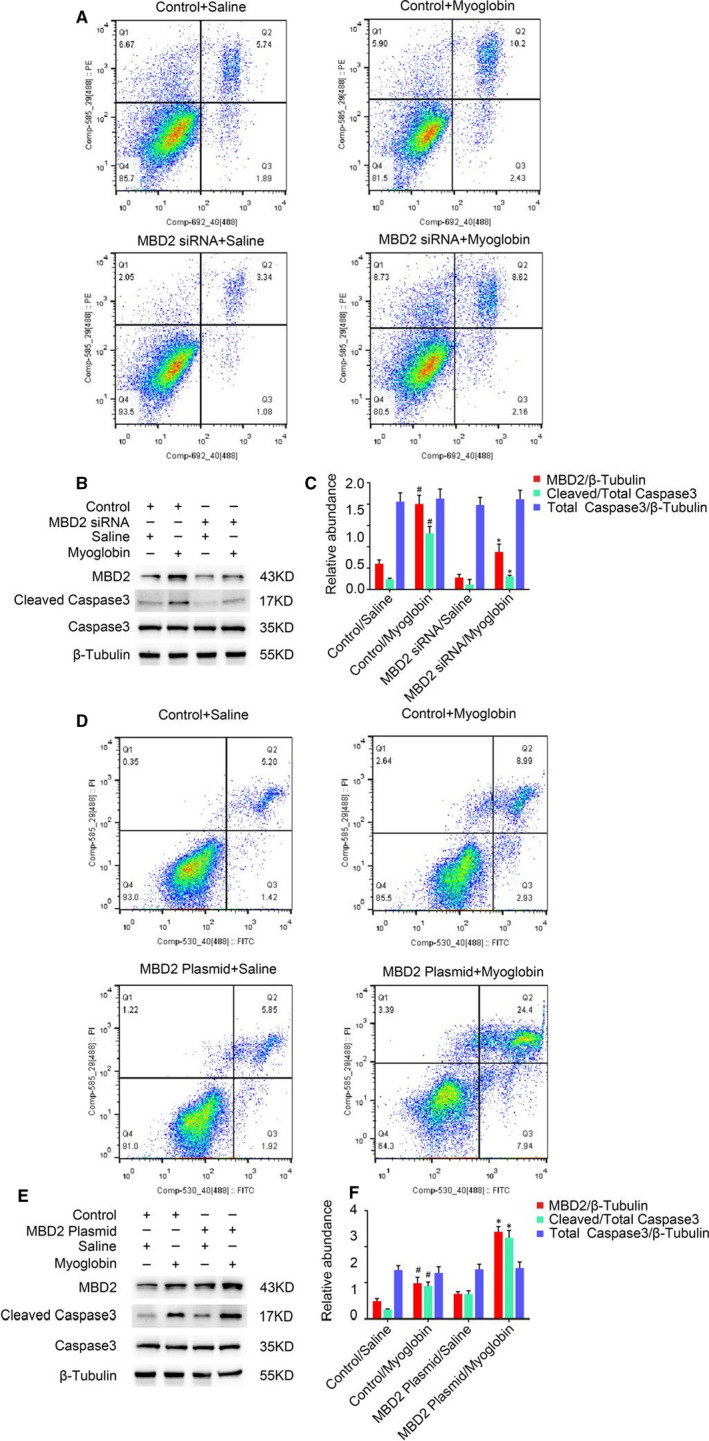
MBD2 mediated myoglobin‐induced renal cell apoptosis in BUMPT cells. BUMPT cells were transfected with 50 nmol/L MBD2 plasmid or MBD2 siRNA, followed by treatment with 200 mM ferrous myoglobin for 6 h. A and D, Flow cytometry analysis of apoptosis in myoglobin‐treated BUMPT cells. B and E, Western blot of MBD2, Caspase‐3, cleaved caspase‐3, and β‐Tubulin expression. C and F, Protein expression levels were quantified by densitometry. Data are expressed as mean ± SD (n = 6). ^#^ *P* < .05 vs Control/Saline group, *P < .05 vs Control/Myoglobin group

As MBD2 expression was up‐regulated as a result of myoglobin treatment in BUMPT cells (Figure 1A,B), we investigated whether MBD2 played a role in renal cell apoptosis in vitro. BUMPT cells were first transfected with *MBD2* siRNA or *MBD2*‐containing plasmid, followed by myoglobin or saline treatment. Flow cytometry analysis demonstrated that the transfection of *MBD2* siRNA reduced apoptosis in BUMPT cells induced by myoglobin (Figure 3A). Immunoblotting analysis showed that *MBD2* siRNA markedly down‐regulated the expression levels of MBD2 and cleaved caspase‐3 (Figure 3B), which was confirmed by densitometric analysis (Figure 3C). In contrast, myoglobin‐treated BUMPT cells containing the *MBD2* plasmid showed increased levels of cleaved caspase‐3 compared with those cells that lacked the plasmid (Figure 3D), indicating that apoptosis was enhanced in myoglobin‐treated cells that ectopically expressed MBD2. This result was further validated by immunoblotting and densitometric analyses (Figure 3E,F).

### 3.4 Tox4 is down‐regulated in MBD2‐KO mice and is involved in renal cell apoptosis

**FIGURE 4:**
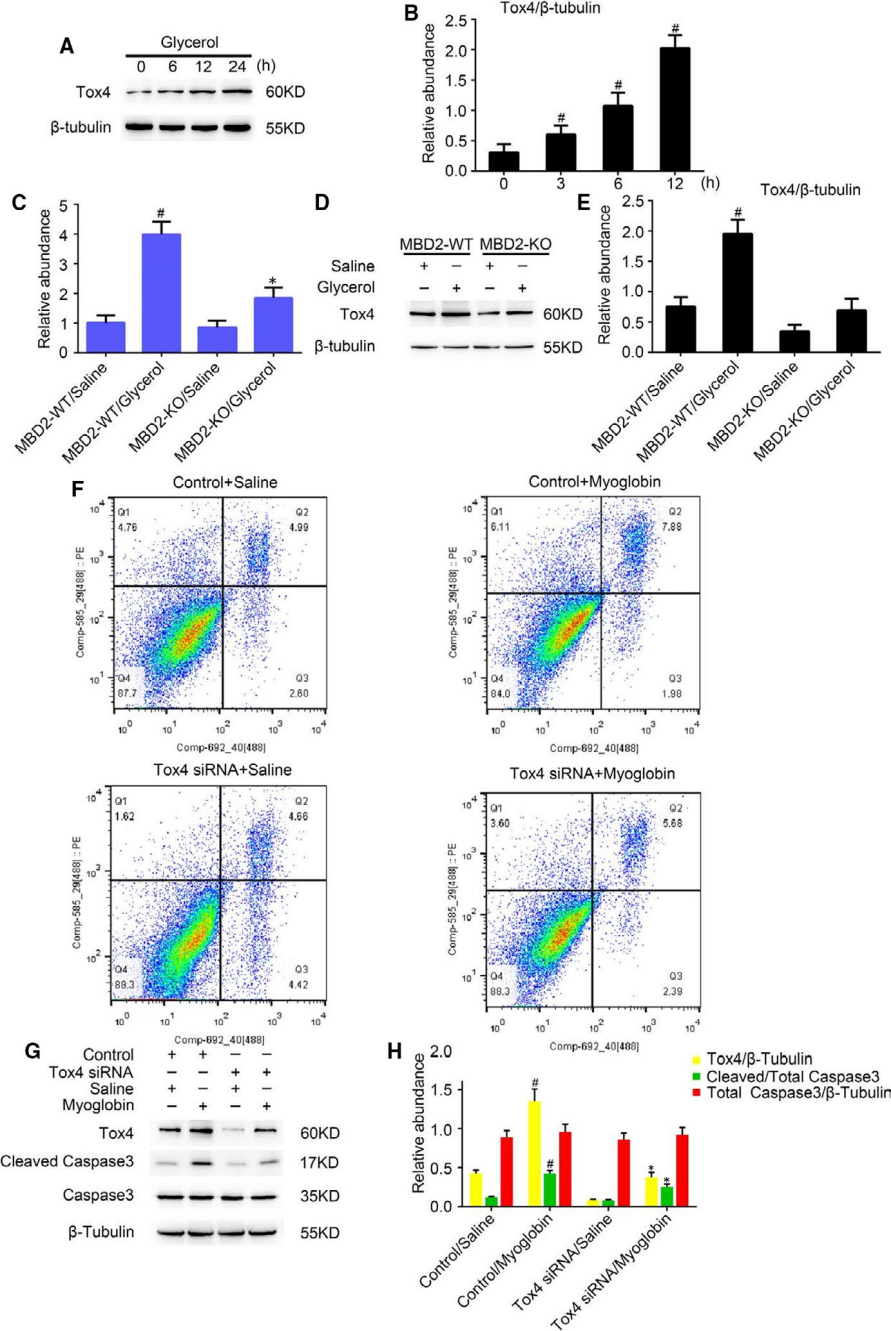
Tox4 expression is inhibited in MBD2‐knockout (KO) mice during RM‐induced AKI, and Tox4 mediated renal cell apoptosis in myoglobin‐treated BUMPT cells. Wild‐type (WT) and MBD2‐KO littermate mice were injected with 8.0 mL/kg glycerol or saline as control and assessed for 24 h. A, Western blot analysis of Tox4 and β‐Tubulin expression over time. B, Densitometric ratios of Tox4/β‐Tubulin over time. C, Real‐time PCR analysis of Tox4 expression. D, Expression levels of Tox4 and β‐Tubulin tested by western blotting in WT and MBD2‐KO mice with and without glycerol treatment. E, Densitometric ratios of Tox4/β‐Tubulin WT and MBD2‐KO mice with and without glycerol treatment. F, Flow cytometry analysis of apoptosis in myoglobin‐treated BUMPT cells. G, Western blot of MBD2, Caspase‐3, cleaved caspase‐3 and β‐Tubulin expression. H, Protein expression levels were quantified by densitometry. Data are expressed as mean ± SD (n = 6). ^#^ *P* < .05 vs MBD2‐WT/Saline group, *P < .05 vs MBD2‐WT/glycerol group in B, C and E. ^#^ *P* < .05 vs Control/Saline group, *P < .05 vs Control/Myoglobin group in G

*Tox4* is novel gene that is reportedly involved in DNA reprogramming, transcription modulation and apoptosis,^22^, ^23^, ^24^ we investigated whether Tox4 was involved in renal cell apoptosis and if MBD2 played a role in *Tox4* regulation during glycerol‐induced AKI. First, we checked Tox4 expression levels in mice during glycerol‐induced AKI. As shown in Figure 4A, Tox4 expression gradually increased over time in WT mouse kidney tissues up to 24 hours after glycerol injection, which was verified by densitometric quantitation (Figure 4B). Real‐time PCR analysis showed that *Tox4* expression was up‐regulated in WT mice compared with MBD2‐KO mice during RM‐induced AKI (Figure 4C), which was confirmed by Western blotting and densitometric analyses (Figure 4D,E). Interestingly, addition of *Tox4* siRNA suppressed apoptosis in myoglobin‐treated BUMPT cells (Figure 4F). These results were also verified by Western blotting, which showed a down‐regulation of cleaved caspase‐3 in myoglobin‐treated BUMPT cells transfected with *Tox4* siRNA compared with control (Figure 4G). Densitometric analysis confirmed the results of western blotting (Figure 4H). Altogether, the results indicated that MBD2 KO resulted in reduced expression of *Tox4* in vivo compared with WT; and inactivation of *Tox4* attenuated BUMPT cell apoptosis in vitro.

### 3.5 MBD2 up‐regulates Tox4 expression by inhibiting methylation of the Tox4 promoter

**FIGURE 5:**
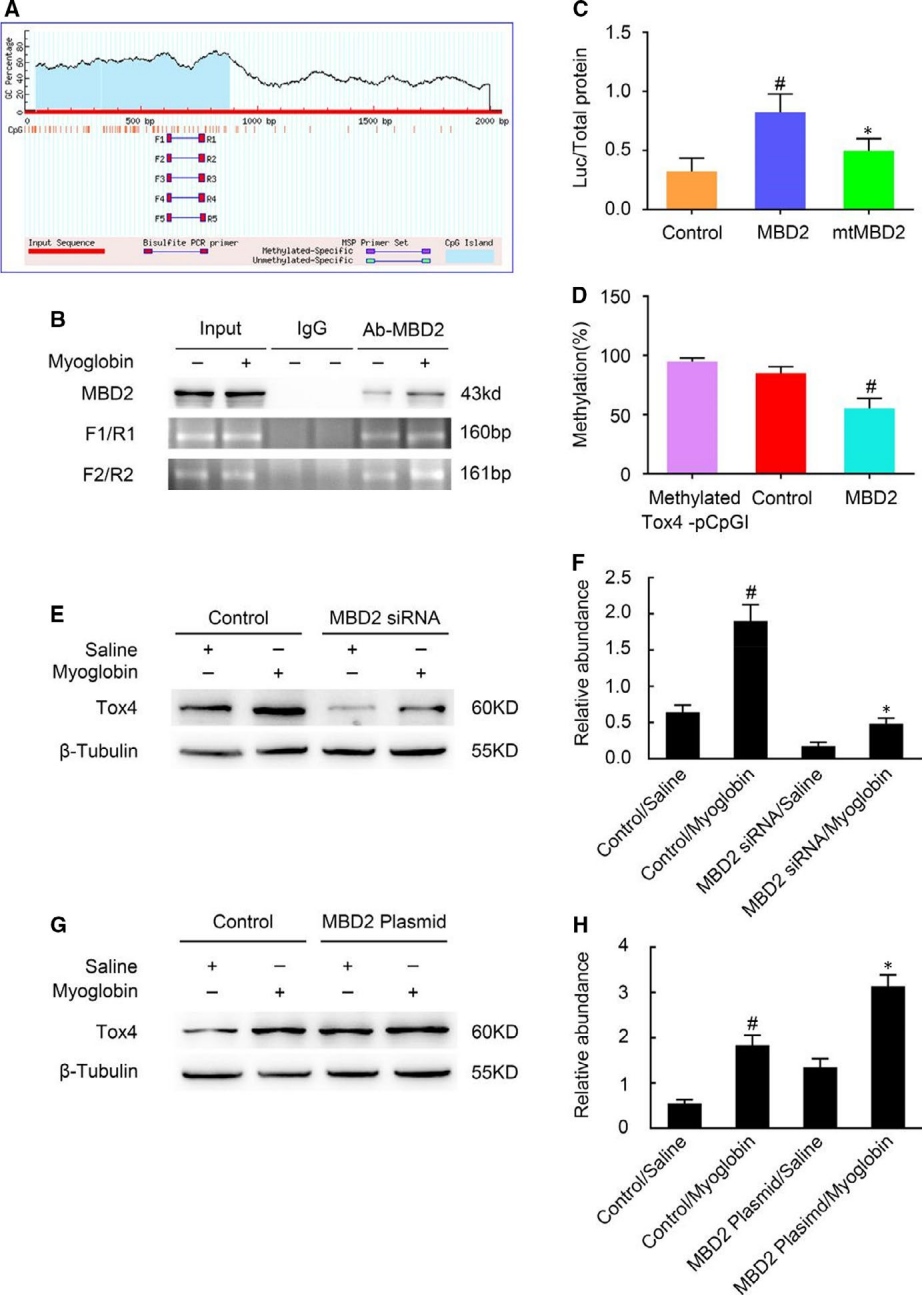
MBD2 directly binds to CpG islands in the promoter of *Tox4* and positively activates transcription via demethylation. A, the presence of CpG islands in the Tox4 promoter and five pairs of primer sequences were predicted by the MethPrimer software. B, Chromatin isolated from BUMPT cells treated with or without myoglobin were used for ChIP assays, which revealed MBD2‐binding sites on the Tox4 promoter. C, Relative luciferase activity of the MBD2 or MBD2 lacking the methylated DNA‐binding domain (mtMBD2) plasmids co‐transfected with the methylated Tox4 pCpGI plasmid in BUMPT cells. D, CpG‐DNA methylation of the Tox4 promoter region. E and G, Expression levels of Tox4 and β‐Tubulin detected by immunoblotting. F and H, Greyscale image analysis of Tox4 levels normalized to β‐Tubulin levels. These data are shown as means ± SD (n = 6). ^#^ *P* < .05 vs Control/Saline group. *P < .05 vs Control/ myoglobin group

We next investigated whether MBD2 regulated Tox4 expression via DNA methylation. *Tox4* has been predicted by MethPrimer to contain a CpG island in its promoter region (http://www.urogene.org/cgi‐bin/methprimer/MethPrimer.cgi), and five pairs of PCR primers for this region have been designed by this website (Figure 5A). ChIP assays showed two binding sites for MBD2 in the *Tox4* promoter region—a 160‐bp fragment (mBS1) and a 161‐bp fragment (mBS2) (Figure 5B), which validated the prediction that MBD2 could directly bind to the *Tox4* promoter region. The *Tox4* promoter region containing the DNA methylation target sequences was cloned into a CpG‐free pCpGI luciferase reporter plasmid and co‐transfected with plasmids containing MBD2 or mutated MBD2 that lacks the DNA‐binding domain (mtMBD2) into BUMPT cells. We found that the MBD2 plasmid markedly activated the transcription level of *Tox4* compared with the plasmid containing mtMBD2 and the control plasmid (Figure 5C). Methylation analysis indicated that endogenous MBD2‐bound DNA markedly suppressed the methylated Tox4 pCpGI; meanwhile, this suppression impact was significantly increased by ectopic MBD2 (Figure 5D). Immunoblot analysis showed that Tox4 expression was up‐regulated by the addition of myoglobin, which was further enhanced by ectopic MBD2 expression (Figure 5G,H). In contrast, MBD2 siRNA treatment suppressed the myoglobin‐induced up‐regulation of Tox4 (Figure 5E,F). Altogether, the results demonstrated that MBD2 enhanced the expression level of Tox4 via promoter demethylation.

## 4 DISCUSSION

RM is often accompanied by AKI caused by crushing trauma.^5^, ^25^ However, due to a lack of knowledge of the underlying mechanisms, no specific therapies for RM‐induced AKI currently exist, thus making it a life‐threatening medical condition. We believe that the present study is the first to show that MBD2 induces renal cell apoptosis in RM‐induced AKI by up‐regulating Tox4 expression via promoter demethylation.

We have previously shown that MBD2 mediates apoptosis during vancomycin‐associated AKI.^26^ In this study, we observed that MBD2 is activated by glycerol in mice and by myoglobin in BUMPT cells, and its expression is largely localized in the nucleus of injured tubular cells (Figure 1). Our results also showed that the deletion of MBD2 could attenuate renal injury and dysfunction in RM‐induced AKI mice. Previous results have indicated that apoptosis plays a key role in the progression of RM‐induced AKI.^16^, ^17^, ^19^, ^20^, ^21^, ^27^ However, few researches have revealed the connection and the underlying mechanism between apoptosis and DNA methylation.^28^ MBD2, a key methylated protein reader, is known to have unique functions that have been shown to contribute to transcriptional regulation in pluripotent cells, immune lymphocytes and in tumorigenesis. Therefore, experiments were carried out and the results showed that glycerol‐induced renal cell apoptosis was significantly attenuated in MBD2‐KO mice in vivo, and myoglobin‐induced BUMPT cell apoptosis was reduced by MBD2 siRNA treatment in vitro, thus indicating an important role for MBD2 in renal cell apoptosis.

**FIGURE 6:**
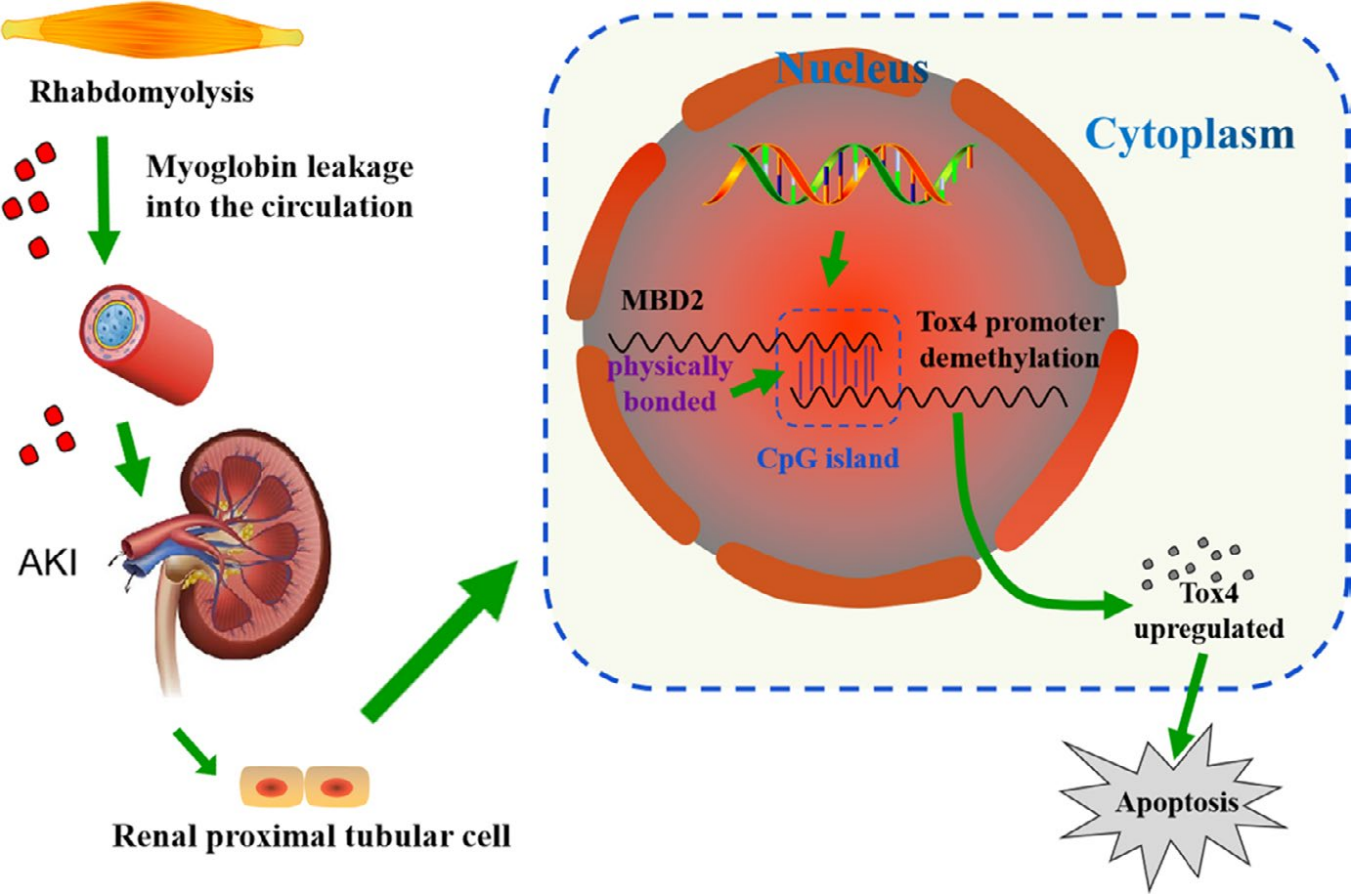
The molecular mechanism of MBD2 in rhabdomyolysis (RM)‐associated acute kidney injury (AKI). When RM occurs, myoglobin is released into the blood, thus inducing nephrotoxicity and, often, AKI. Subsequently, MBD2 activates the expression of Tox4 via demethylation of its promoter, which mediates renal cell apoptosis

The above findings revealed that MBD2 plays a pivotal role in this model; however, the molecular mechanism of this role required further investigation. Therefore, we assessed the changes in downstream gene expression in response to glycerol injection. Both PCR and immunoblot results indicated that the expression level of Tox4 was increased in WT mice during RM‐induced AKI and reduced in MBD2‐KO mice (Figure 4A‐E). The functional analysis indicated that Tox4 positively regulated cell apoptosis and the protein expression level of cleaved caspase‐3 confirmed this conclusion (Figure 4F‐H). Thus, inhibition of Tox4 ameliorates myoglobin‐induced renal cell apoptosis suggesting that Tox4 may be a potential therapeutic target for RM‐associated AKI. We also found that the expression of Tox4 was up‐regulated following transfection of the MBD2 plasmid, whereas it was decreased in response to the MBD2 siRNA treatment (Figure 5E‐H). The ChIP assays demonstrated that MBD2 could directly interact with a binding site within the Tox4 promoter domain it (Figure 5B). Moreover, observed that MBD2 activated Tox4 via demethylation (Figure 5C,D). These results confirmed our hypothesis that Tox4 is a direct downstream target gene of MBD2. In recent studies, Tox4 was identified as a novel transcriptional modulator,^22^ but the impact of Tox4 on apoptosis in this experimental model remained poorly understood. In order to explore the regulatory mechanism of Tox4 in RM‐associated AKI, we transfected Tox4 siRNA synthesized by the Ruibo RUIBO Biology company Co. in BUMPT cells. As shown in Figure 6, our results verified that Tox4 promoter had a direct interaction with MBD2 in this experimental model, which up‐regulated TOX4 transcription level and its protein expression. However, we have not found the interactions between Tox4 protein and other apoptotic proteins or DNA fragments, which need to be further explored in the future work.

In conclusion, our results indicated that MBD2 mediates apoptosis during RM‐associated AKI. In addition, we found that MBD2 regulates the expression of Tox4 via promoter demethylation to induce renal cell apoptosis. Therefore, MBD2 may potentially be a new therapeutic target for treating RM‐induced AKI.

## CONFLICT OF INTEREST

The authors declare that the research was conducted in the absence of any commercial or financial relationships that could be construed as a potential conflict of interest.

## AUTHOR CONTRIBUTION

**Tianshi Sun:** Data curation (equal); formal analysis (equal); investigation (equal); methodology (equal); project administration (equal); resources (equal); software (equal); supervision (equal); validation (equal); visualization (equal); writing – original draft (equal); writing – review and editing (equal). **Qing Liu:** Formal analysis (equal); investigation (equal); software (equal); supervision (equal); writing – review and editing (equal). **Yifan Wang:** Investigation (equal); methodology (equal); software (equal). **Youwen Deng:** Conceptualization (equal); funding acquisition (equal); supervision (equal); writing – review and editing (equal). **Dongshan Zhang:** Conceptualization (equal); methodology (equal); resources (equal); writing – review and editing (equal).

## CONSENT FOR PUBLICATION

Figures in the manuscript have been published with the consent of all the authors.
